# Robot-Assisted Partial Nephrectomy Mid-Term Oncologic Outcomes: A Systematic Review

**DOI:** 10.3390/jcm11206165

**Published:** 2022-10-19

**Authors:** Mihai Dorin Vartolomei, Mesut Remzi, Harun Fajkovic, Shahrokh F. Shariat

**Affiliations:** 1 Department of Urology, Comprehensive Cancer Center, Medical University of Vienna, 1090 Vienna, Austria; 2 Institution Organizing University Doctoral Studies IOSUD, George Emil Palade University of Medicine, Pharmacy, Sciences and Technology, 540142 Târgu Mureș, Romania; 3 Institute for Urology and Reproductive Health, I.M. Sechenov First Moscow State Medical University, Moscow 119992, Russia; 4 Department of Urology, Weill Cornell Medical College, New York, NY 14853, USA; 5 Department of Urology, University of Texas Southwestern, Dallas, TX 75390, USA; 6 Karl Landsteiner Institute of Urology and Andrology, 1090 Vienna, Austria; 7 Department of Urology, Second Faculty of Medicine, Charles University, 15006 Prague, Czech Republic; 8 Hourani Center for Applied Scientific Research, Al Ahlizza Amman University, Amman 19328, Jordan

**Keywords:** partial nephrectomy, renal cancer, metastasis, overall survival, robot-assisted, cancer-specific survival, nephron-sparing surgery

## Abstract

Background: Robot-assisted partial nephrectomy (RAPN) is used more and more in present days as a therapy option for surgical treatment of cT1 renal masses. Current guidelines equally recommend open (OPN), laparoscopic (LPN), or robotic partial nephrectomy (PN). The aim of this review was to analyze the most representative RAPN series in terms of reported oncological outcomes. (2) Methods: A systematic search of Webofscience, PUBMED, Clinicaltrials.gov was performed on 1 August 2022. Studies were considered eligible if they: included patients with renal cell carcinoma (RCC) stage T1, were prospective, used randomized clinical trials (RCT) or retrospective studies, had patients undergo RAPN with a minimum follow-up of 48 months. (3) Results: Reported positive surgical margin rates were from 0 to 10.5%. Local recurrence occurred in up to 3.6% of patients. Distant metastases were reported in up to 6.4% of patients. 5-year cancer free survival (CFS) estimates rates ranged from 86.4% to 98.4%. 5-year cancer specific survival (CSS) estimates rates ranged from 90.1% to 100%, and 5-year overall survival (OS) estimates rated ranged from 82.6% to 97.9%. (4) Conclusions: Data coming from retrospective and prospective series shows very good oncologic outcomes after RAPN. Up to now, 10-year survival outcomes were not reported. Taken together, RAPN deliver similar oncologic performance to OPN and LPN.

## 1. Introduction

Renal cell carcinoma (RCC) represents a serious condition and its incidence in the US alone is estimated to 79,000 new cases in 2022 [1] and worldwide up to half a million [2]. For small renal masses (cT1) and some larger lesions (cT2), partial nephrectomy (PN) has established itself as the preferred surgical diagnostic and therapeutic strategy [3,4,5].

With the advent of robotic surgery, robot-assisted partial nephrectomy (RAPN) has become an increasingly used surgical option for the surgical management of cT1 and some cT2 renal masses [6]; it is standardized [7] and can be performed both transperitoneal or retroperitoneal [8,9].

Current guidelines [10] equally recommend open (OPN), laparoscopic (LPN), or robotic partial nephrectomy (PN), as a head-to-head comparison in randomized trials have not been performed until now, and are unlikely to be ever performed. A recent meta-analysis, including seven studies with 2646 patients, that compared RAPN to OPN, showed that RAPN resulted in less estimated blood loss, shorter length of hospital stay, and fewer complications; but it was lacking data on oncologic outcomes [11]. While the most recent systematic review showed slightly higher cancer-specific survival (CSS) rates for RAPN, with a 5-year CSS of 90.1–97.9% for RAPN versus 85.9–86.9% for LPN and 88.5–96.3% for OPN, when only studies with matched-pair analysis were included [12]. 

Considering the continuous evolution in current evidence, we aimed to analyze the most representative RAPN series in terms of reported oncological outcomes.

## 2. Materials and Methods

A systematic search of Webofscience, PUBMED, Clinicaltrials.gov was performed on 1 August 2022, using any combination of the terms: robot-assisted (EXP) OR partial nephrectomy (EXP) OR robot-assisted partial nephrectomy (EXP) AND renal carcinoma (EXP) OR kidney cancer (EXP). All original articles that fulfilled the inclusion criteria were included. Supplementary search of gray literature was performed in Google scholar.

### 2.1. Protocol

The protocol of this systematic review followed the Cochrane handbook [13] and the Preferred Reporting Items for Systematic Reviews and Meta-analysis (PRISMA) criteria (www.prisma-statement.org, accessed on 1 August 2022) [14]. 

### 2.2. Inclusion and Exclusion Criteria

Studies were considered eligible if they included patients with RCC, stage T1, had a prospective, randomized clinical trial (RCT) or retrospective design, patients underwent RAPN, had a mean follow-up of 48 months, and reported oncologic outcomes for five years. 

Primary outcome was cancer-free survival (CFS). Secondary outcomes were overall survival (OS) and CSS. 

For each selected study, the following items were recorded: first author’s name, year of publication, design, number of patients, age, tumor size on imaging, rate of positive surgical margins (PSM), local recurrence, distant metastasis, CFS, OS, CSS, and follow-up. Two investigators independently conducted literature searches and extracted data from included full-text articles; disagreements were resolved by consensus.

## 3. Results

After fulfilling all the inclusion criteria, 13 studies were identified [15,16,17,18,19,20,21,22,23,24,25,26,27], and another 17 studies were excluded mainly because follow-up was too short [28,29,30,31,32,33,34,35,36,37,38,39,40,41,42,43,44]. All of the studies had an retrospective design except the ones from Furukawa et al. [16] and Beauval et al. [24]. The studies included in total 2703 patients with T1 RCC. The mean age of the patients ranged from 49.8 to 61 years. Mean tumor size ranged from 2.6 to 4.2 cm. Follow-ups ranged from 49 to 88, and only 4 studies had a shorter than 60 month follow-up [17,21,22,25]. Reported positive surgical margin rates ranged from 0 to 10.5%. Local recurrence was reported in up to 3.6% of patients [20]. Distant metastases were reported in up to 6.4% of patients [24]. 5-year CFS estimates rates ranged from 86.4% to 98.4%. 5-year CSS estimates rates ranged from 90.1% to 100%, and 5-year OS estimates rated ranged from 82.6% to 97.9% (Table 1).

## 4. Discussion

We found that RAPN resulted in very good oncological outcomes. Despite these promising results, they came quite late, as RAPN was first described almost two decades ago [45]; to date, long-term (10 years) oncologic outcomes are not reported yet. Seventeen studies were excluded, as we included in this review only the most relevant studies with a minimum of four years of follow-up and reported oncologic outcomes at five years (CFS, OS and/or CSS). Even so many studies are retrospective, and some may include also patients treated while on learning curve. That could explain the heterogeneous results regarding the rate of PSM, with rates ranging from 0 to 10.5%; this certainly negatively impacts oncologic outcomes. Indeed, a recent meta-analysis including 44 studies assessing PN patients demonstrated that PSM increased the risk of local recurrence (RR 4.14 95% CI 2.75–6.24), all site recurrence (RR 4.8 95% CI 3.38–6.62), mortality (RR 1.83 95% CI 1.08–3.1), and metastasis (RR 8.1 95% CI 3.88–16.92) [46]. However, research has shown that recurrence within 5 years of nephrectomy is a strong surrogate for long-term outcomes, including OS and CSS [47,48].

The local recurrence rate was, in agreement with the literature, up to 3.6% [49]. Further, the distant metastasis rate was acceptably low, with a maximum reported rate of 6.4% in a prospective multicenter study [24]; this rate is also in line with those reported by historic open and laparoscopic PN series [50]. 

Oncologic endpoints, such as CFS, CSS, or OS, were very good, but with a large range, such as approximately 12% for CFS, 10% for CSS, and 15% for OS this may be due to heterogeneity in follow-up, characteristics of included patients, surgeon experience, and natural history of RCC. Nevertheless, these mid-term outcomes are similar to those reported in open or laparoscopic series [51]. A head-to-head prospective comparison had, until now, not been performed, and is unlikely to ever be performed. Recently Audigé et al. showed, in a retrospective analysis, that CFS and OS did not differ at seven years of follow-up; however a greater amount of local recurrences were reported in the OPN cohort [15]. Other comparison studies with minimum four years of follow-up reported slightly better CFS, CSS or OS in favor of RAPN over OPN [22,23,25]; however, lack of standardized reporting of results, heterogeneity of the studies, and patient selection may have influenced the results.

## 5. Conclusions

Data coming from retrospective and prospective series show very good oncologic outcomes after RAPN. While 10-year survival outcomes are not reported yet, oncologically, it is highly likely that RAPN performs equally to LPN and OPN when performed by experienced surgeons. Short- and long-term differences where RAPN is likely to outperform LPN and/or OPN are the surgical and perioperative quality indicators, and may be health related quality of life domains.

## Figures and Tables

**Table 1:** Robot assisted partial nephrectomy series with 5 year reported oncologic outcomes.

Study/Year	Design	No. of Patients	Mean Age	Mean Size CT (cm)	Follow-Up (Months)	PSM, No. (%)	Local Rec., No. (%)	Distant Metastases, No. (%)	CFS	OS	CSS
Audigé et al., 2022 [15]	Retro RAPN vs. OPN	162/167	59.1/62.2	2.96/3.18	85/162	2 (1)/ 12(5.9)	3 (1.8)/11 (6.5)	3 (1.8)/ 8 (4.8)	5-year aprox. 85% in both groups	5-year aprox. 90% in both groups	NA
Furukawa et al., 2022 [16]	Prospective multicenter	103	61	2.7	60	0%	0%	5 (4.9)	92.8%	97.9%	100%
Koukourikis et al., 2021 [17]	Retro.	155	53	4.2	58	15 (10.5)	3 (2.1)	4 (2.8)	93.6%	96.7%	NA
Carbonara et al., 2021 [18]	Retro. multicenter	85	58	3	88	7 (8.2)	0%	2 (2.33)	91.7%	91.7%	97.7%
Kızılay et al., 2019 [19]	Retro. RAPN vs. LPN	71/71	52.9/54.6	2.48/2.79	58.1/64.8	2 (2.8) 3 (4.2)	NA	2 (2.8) 3 (4.2)	97.1%/ 95.8%	82.6%/ 84.8%	90.1%/ 85.9%
Bertolo et al., 2019 [20]	Prospectively-maintained database	278	60.1	3.24	70.8	12 (4.3)	3.61%	3.24%	NA	89.9%	98.2%
Vartolomei et al., 2019 [21]	Retro.	90	61	3	59	2 (2.2)	2 (2.2)	5 (5.5)	97.5%	95.1%	97.5%
Ali Abdel Raheem et al., 2019 [22]	Retro. RAPN vs. OPN	52/37	49.8/ 50.9	2.8/2.5	59/53	5 (9.6)/ 3 (8.1)	1 (2.3) 2 (5.9)	1 (2.3) 1 (2.9)	94%/ 95.8%	95.5%/ 93.8%	100%/ 93.8%
Chang et al., 2018 [23]	Retro. RAPN vs. LPN vs. OPN	1,308,380/ 206/ 722	53.2/ 53.5/ 53.8	2.8/2.7/2.5	60/ 60/ 64	3 (2.5)/ 5 (4.1)/2 (1.6)	NA	NA	98.4%/ 99.2%/ 98.4%	NA	90.2%/ 86.9%/ 88.5%
Beauval et al., 2018 [24]	Prospective multicenter	110	61	3	64.4	11/10	3/(2.7)	7/(6.4)	86.4%	94.9%	96.8%
Wang et al., 2016 [25]	Retro. RAPN vs. OPN	164/159	61.8/59.8	3.8/3.6	49/52	8 (5)	6 (3.7) 3 (1.8)	2 (1.2) 3 (1.9)	95.1%/ 92.7%	NA	98.7%/ 97.6%
Andrade et al., 2016 [26]	Retro.	110	59.8	2.6	61.9	2 (1.7)	0%	2 (1.7)	97.8%	91.1%	97.8%
Khalifeh et al., 2013 [27]	Retro. multicenter	943	59	2.9	63.6	21 (2.2)	9 (1) 94.8%	4 (0.4) 97.5%	96%	88%	99.2%

CFS, cancer-free survival; CSS, cancer-specific survival; OPN, open partial nephrectomy; OS, overall survival; PSM, positive surgical margins; RAPN, robot-assisted partial nephrectomy; Retro, retrospective; CT: computer tomography; NA: not available.

## Data Availability

Not applicable.
